# Daily cycle in oxygen consumption by the sea anemone *Nematostella vectensis* Stephenson

**DOI:** 10.1242/bio.013474

**Published:** 2016-01-15

**Authors:** Amy E. Maas, Ian T. Jones, Adam M. Reitzel, Ann M. Tarrant

**Affiliations:** 1 Biology Department, Woods Hole Oceanographic Institution, Woods Hole, MA 02543, USA; 2 Bermuda Institute of Ocean Sciences, St. George's GE01, Bermuda; 3 School of Marine Sciences, University of Maine, Orono, ME 04469, USA; 4 Department of Biological Sciences, University of North Carolina at Charlotte, Charlotte, NC 28223, USA

**Keywords:** Circadian, Cnidarian, Respirometry

## Abstract

In bilaterian animals, the circadian clock is intimately involved in regulating energetic metabolism. Although cnidarians exhibit diel behavioral rhythms including cycles in locomotor activity, tentacle extension and spawning, daily cycles in cnidarian metabolism have not been described. To explore a possible circadian metabolic cycle, we maintained the anemone *Nematostella vectensis* in a 12 h light/dark cycle, a reversed light cycle, or in constant darkness. Oxygen consumption rates were measured at intervals using an optical oxygen meter. Respiration rates responded to entrainment with higher rates during light periods. During a second experiment with higher temporal resolution, respiration rates peaked late in the light period. The diel pattern could be detected after six days in constant darkness. Together, our results suggest that respiration rates in *Nematostella* exhibit a daily cycle that may be under circadian control and that the cycle in respiration rate is not driven by the previously described nocturnal increase in locomotor activity in this species.

## Abstract (precis)

**Summary:** This study demonstrates that the sea anemone *Nematostella* exhibits a daily cycle in respiration rate that is not driven by the previously described nocturnal increase in movement in this species.

## INTRODUCTION

Circadian rhythms are biological cycles that oscillate with a period of about 24 h. While they are typically entrained by environmental cues, such as cycles in light or temperature, circadian rhythms also persist under constant ‘free-running’ conditions. In animals, behavioral and physiological rhythms are driven by transcriptional and translational cycles in the underlying molecular components of the clock. Studies in diverse animals have demonstrated roles of molecular circadian cycles in regulating metabolism, including effects on feeding behavior, carbohydrate and lipid metabolism, and xenobiotic detoxification (Dibner and Schibler, 2015; Migliori et al., 2011). In turn, feeding rhythms entrain circadian cycles, particularly within organs and tissues regulating these processes, such as the mammalian liver, intestine and kidney.

The molecular architecture underlying the circadian clock is relatively well conserved in animals, sharing similar protein-protein interactions that involve central, positive elements called CLOCK and CYCLE, which heterodimerize together and bind to specific sequence motifs to regulate transcription of clock-controlled genes. Well documented in mammals and insects, homologs of these transcription factors have also been found in cnidarians, including corals and the sea anemone *Nematostella vectensis* Stephenson, suggesting ancient origin of circadian feedback loops (Reitzel et al., 2013; Vize, 2009). In addition, transcriptional profiling studies conducted in *Nematostella* and various coral species have shown diel and/or circadian expression patterns of numerous other genes, including those for light signaling and circadian clock regulation (e.g. cryptochromes) as well as metabolic pathways (Brady et al., 2011; Hemond and Vollmer, 2015; Levy et al., 2011; Oren et al., 2015; Ruiz-Jones and Palumbi, 2015). The conserved nature of this pathway indicates that aspects of the circadian clock date to at least the cnidarian-bilaterian ancestor, over 500 million years ago.

While progress has been made in identifying conserved molecular components of the circadian clock, little is known regarding the regulatory roles of circadian clocks among cnidarians. Many cnidarians exhibit diel patterns in tentacle extension, spawning, and other behaviors, but in many groups, most notably reef-building corals, circadian studies are complicated by the presence of photosynthetic algal symbionts. Because *Nematostella* lacks algal symbionts and is amenable to laboratory manipulations, it has emerged as a useful model species for studies of cnidarian physiology and circadian signaling. Indeed, circadian rhythms in locomotor activity have recently been described in *Nematostella* (Hendricks et al., 2012; Oren et al., 2015). In many animals, daily periods of increased movement are associated with periods of elevated oxygen consumption; however, few such measurements have been made in largely sedentary animals. In addition, it is unknown whether cnidarians exhibit a circadian metabolic rhythm, but because many cnidarian species are predominately or exclusively sedentary, these taxa could be insightful for measuring light-entrained changes in metabolism. To address this uncertainty we conducted two experiments to determine whether respiration rate in *Nematostella* varies across light/dark cycles and whether such a rhythm would persist under constant darkness or with altered feeding times.

## RESULTS AND DISCUSSION

To measure oxygen consumption rates, anemones were placed in glass syringes that each contained an oxygen-sensitive optical spot (details in Materials and Methods). Animal respiration rates typically decrease as oxygen concentrations drop to physiologically stressful levels (Guppy and Withers, 1999); however, *Nematostella* is a sediment-dwelling invertebrate that naturally experiences a wide range of oxygen concentrations and may be tolerant of relatively low oxygen levels. Preliminary experiments showed that respiration rate remained constant over periods of 8-11 h (i.e. within a light or dark period), indicating that *Nematostella* is capable of maintaining a continuous respiration rate down to 25% saturation (68 µmol l^−1^ O_2_ at 18°C; Fig. S1). Only 5% of the final oxygen measurements fell below 40% saturation during subsequent experiments, and all of these were above 25% saturation, suggesting that response to hypoxia did not affect our observations.

**Fig. 1.**
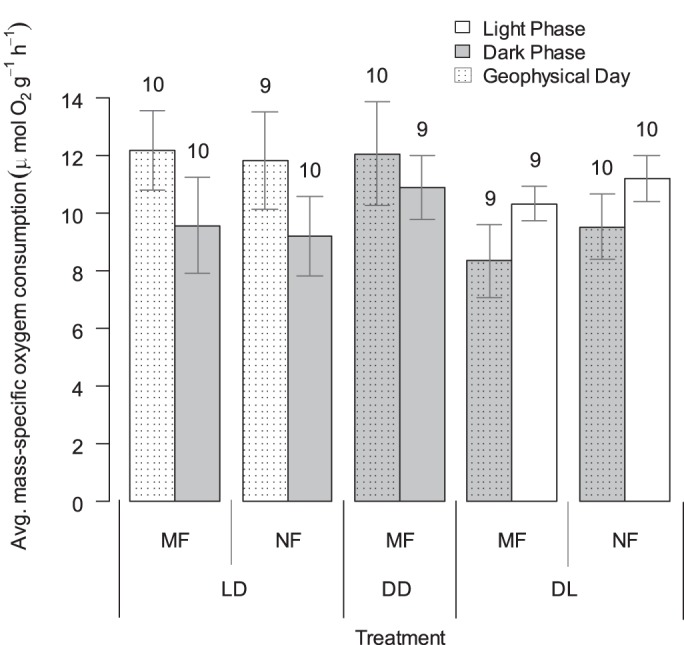
**The respiration rate of *Nematostella* was higher during light periods and not affected by geophysical time or time of feeding.** Dry mass-specific respiration rates of anemones in five treatments that included combinations of three light treatments (LD: normal 12:12 h light-dark cycle, DL: reversed light cycle with darkness during the day, DD: constant darkness) and two feeding treatments (MF: fed during morning, NF: fed at night). Error bars represent ±s.e.m. (*n*=9-10 individuals per group, labels above bars). Oxygen consumption rates were higher during light periods within both the DL and LD treatments, regardless of feeding time (see text and Table S1 for further detail).

In a first experiment to examine daily cycles in *Nematostella* respiration rates, anemones were maintained for two weeks in one of three different light treatments: a normal 12 h light-dark cycle (LD), a 12 h reversed dark-light cycle (DL), or continuous darkness (DD). To test for possible effects of temporally-restricted feeding on energetic metabolism, animals within the LD and DL cycles were fed either in the morning (MF) or at night (NF); all animals within the DD treatment were fed in the morning. Oxygen consumption rates were then measured for animals within each group over 4-h periods beginning at noon (i.e. ZT5 for LD, ZT17 for DL; ZT indicates zeitgeber time) and midnight (i.e. ZT17 for LD, ZT5 for DL) (Fig. 1). The respiration rate of individuals in the DD treatment was not significantly affected by the time of day (*P*=0.917). In addition, the respiration rate of individuals in the LD and DL treatments was not affected by the time of feeding (*P*=0.535, full statistical results in Table S1). Together these results indicate that food availability did not provide a strong entraining signal and that the energetic cost of food capture and processing do not drive significant differences in metabolic rate in *Nematostella*. There was, however, a significant effect of the zeitgeber time (light vs dark periods, *P*=0.0152), with both the LD and DL groups exhibiting higher oxygen consumption rates during light periods (ZT5-9) relative to dark periods (ZT17-23). In contrast, there was no consistent effect of time of day (geophysical time, *P*=0.918), indicating that it is possible to entrain *Nematostella* to a reversed light cycle and shift the diel pattern in oxygen consumption rate, consistent with previous work on behavioral shifts in response to advanced light cycles (Hendricks et al., 2012). While the plot of mass-specific respiration rates (Fig. 1) suggests trends toward reduced respiration under DL conditions relative to LD and a daily cycle with higher daytime respiration under constant darkness, these apparent trends were not statistically significant in analysis of covariance (ANCOVA). Individuals maintained in constant darkness showed no statistical difference in respiration rate between day and night (*P*=0.917), suggesting that any free-running rhythm was lost within the two-week habituation period.

**Fig. 2.**
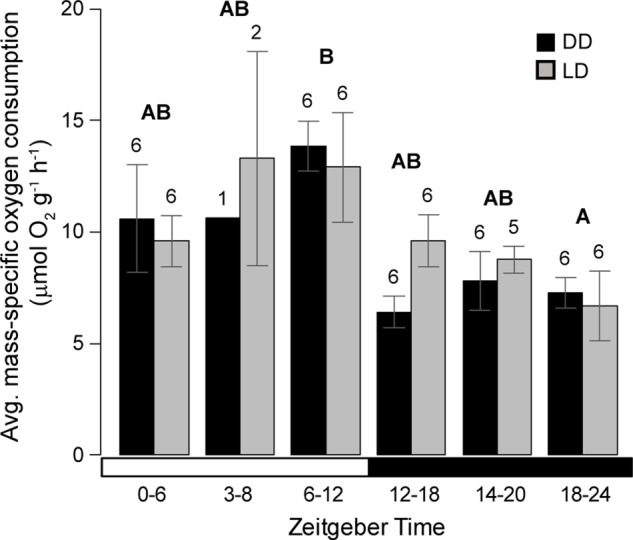
***Nematostella* is able to maintain a metabolic rhythm after six days in DD conditions.** Dry mass-specific respiration rate of anemones over six time points under DD (constant darkness) and LD (12:12 h light-dark cycle) conditions. Error bars represent ±s.e.m. Numbers above bars indicate sample sizes. Different letters above bars represent statistically different means (combining data from DD and LD treatments at a given time interval) using a Tukey's post-hoc test. The bottom bar represents light time periods (white) and dark time periods (black).

A second experiment was designed to provide a higher temporal resolution of the cyclic pattern in *Nematostella* oxygen consumption. Animals were maintained in LD or DD conditions for six days with morning feedings. Respiration rates were then measured at six partially overlapping time intervals throughout a 24-h period. In this experiment, respiration rates varied significantly over the day (*P*=0.0025, full statistical results in Table S2) with maximum observed rates during the latter part of the light period (ZT6-12, 13:00-19:00; Fig. 2). The patterns of respiration rate in the dark were remarkably similar and not statistically different between the LD and DD treatments (*P*=0.574), suggesting the presence of a free-running rhythm that persisted over six days of constant conditions. While feeding was not shown to have a significant effect on metabolism in the previous experiment, the timing for introduction of food was not independently manipulated in this second experiment. Therefore, an alternative explanation is that the pattern observed under constant darkness resulted from entrainment by morning feeding or some other factor that was not accounted for. Together the two experiments show that, *Nematostella* was able to maintain a metabolic rhythm after six days in DD conditions (Fig. 2), but no significant rhythm was detected after 14 days (Fig. 1). Similarly, time-course studies by Peres et al. (2014) show that rhythmic expression patterns of most genes is lost within four days under free-running conditions. Consequently, our metabolic studies are consistent with the gene expression studies in showing that *Nematostella* is able to maintain a rhythm for several days under constant light conditions, but requires environmental entrainment over longer periods.

In addition to the loss of a cycle under extended DD conditions, the amplitude of the daily cycle differed between the two experiments. Under LD conditions, the daytime respiration rates were similar, but nighttime respiration decreased by an average of 19% and 34% in the first and second experiments, respectively. One possibility is that this difference is related to allometric effects on circadian physiology. Within an experiment, animals were of similar size, but animals in the second experiments were larger overall. Size is likely to affect investment in reproduction and may also affect activity patterns, both of which are known to be affected by light cycles in *Nematostella* (Genikhovich and Technau, 2009; Hendricks et al., 2012; Stefanik et al., 2013; Oren et al., 2015).

Both experiments demonstrated higher respiration rates during light periods, and in the first experiment this is independent of feeding time. Higher daytime metabolism contrasts results from previous studies of behavioral rhythms, which showed that *Nematostella* has greater locomotor activity at night (Hendricks et al., 2012; Oren et al., 2015). While studies conducted in other invertebrates usually show a positive correlation between daily patterns in locomotor activity and energetic metabolism, these rhythms are typically studied in relatively active animals, such as fishes (Shekk, 1986) and decapod crustaceans (Aguzzi et al., 2003). Thus, the discrepancy between the behavioral and metabolic observations may be a consequence of the lifestyle of *Nematostella*; being infaunal suspension feeders, it may be that movement is not a major part of their energetic budget. Similarly, *C. elegans* exhibits asynchrony between behavioral and metabolic rhythms, with a dawn peak in pharyngeal muscle contraction and midnight peak in oxygen consumption (Migliori et al., 2011). Other processes that may vary over circadian cycles and influence respiration rate include cell division, growth, repair and regeneration, protein and DNA synthesis, and gametogenesis. Consistent with this, a recent transcriptional profiling study conducted with *Nematostella* has identified daily rhythms in genes needed to prevent and repair damage to DNA and proteins, and a daytime peak in expression of fructose-1,6-bisphosphatase, the rate-limiting enzyme for gluconeogenesis (Oren et al., 2015). Together, studies of gene expression and respirometry suggest that *Nematostella* physiology varies on a circadian cycle and that energetic metabolism is not strictly driven by a rhythm in locomotor activity.

## MATERIALS AND METHODS

### Animal model

*Nematostella vectensis* were originally collected from Sippewissett Marsh in Falmouth, Massachusetts. Animals used in experiments were derived from stock maintained in the laboratory for at least three years and several generations. Individuals were of mixed sexes and were at least six months old.

### *Nematostella vectensis* culture

Prior to experiments, anemones were maintained in a common culture for several months in a walk-in incubator at 18°C in glass dishes containing filtered seawater (15-16 ppt). A 12:12 h light-dark cycle was provided by full-spectrum bulbs (15 W, Zoo Med Reef Sun 50/50 bulbs). Anemones were fed brine shrimp five times a week, and water was changed weekly.

### Experimental design

For the first experiment, anemones were haphazardly distributed among glass dishes (five per dish) to ensure a similar distribution of sizes in each. Four dishes were randomly assigned to each of five groups that were exposed to different light and feeding conditions. Preliminary experiments showed that when offered food, anemones usually began feeding, with no apparent differences between day and night (data not shown).

Animals were maintained either on a normal 12:12 h light-dark cycle (LD conditions, lights on at 07:00=ZT0), on a reversed light cycle (DL, lights on at 19:00=ZT0), or in continuous darkness (DD). Animals within the LD and DD treatments were fed freshly hatched brine shrimp in either the morning (MF, 08:00, ZT1 for LD and ZT13 for DL) or the night (NF, 20:00). Individuals in DD were fed at 08:00 (MF) only. Glass dishes were placed into three water bath bins (one bin per light treatment). Bins were covered with opaque lids at all times except during feeding and water changes. The dishes within each treatment were haphazardly rearranged every two days. Light was provided by full-spectrum bulbs on timers within the bins. Heat produced by the lights generated a day-night temperature difference of approximately 0.6°C in the LD and DL treatments; in comparison temperature fluctuated by about 0.3°C within the DD treatment with no daily cycle. Anemones were fed 4-6 days a week, and water was changed every 3-5 days during light periods. Water changes and feeding that occurred during the dark periods were done using a red light (25 W); this indirect exposure lasted less than 10 min. After two weeks of this maintenance, anemones were starved for three days to allow for gut clearance. Respiration rate was measured (as described below) from either 12:00-16:00 (i.e. ZT5 and ZT17 for LD and DL, respectively) or 0:00-4:00. For logistical reasons, the measurements were spread across two days, with half of the animals in each group measured on each day. No differences were noted between days, so measurements were pooled within groups.

A second experiment was conducted to more finely resolve the timing of an apparent metabolic rhythm. For this, anemones were randomly distributed between LD and DD light cycles and were all fed at approximately 09:00, five days per week. During a six-day habituation period, individuals were exposed to LD conditions through overhead full-spectrum lights (18°C, not affected by light cycle). Dishes containing animals in the DD group were wrapped in aluminum foil to prevent light exposure. As previously, animals were starved for three days prior to respiration measurements. Within a 24-h period, respiration rates were measured from ZT 0-6, 3-8, 6-12, 12-18, 14-20, and 18-0, where ZT0 was 07:00.

### Respirometery

Spots were cut from oxygen sensitive optical foil (OXFOIL, provided by Pyro Sciences, Aachen Germany) and glued to the inner surface of 20 ml gas-tight glass syringes. The optical foil is similar to commercially available OXP5 optical spots (Pyrosciences), but is more flexible, allowing better attachment to the curved inner surface of the syringe. Animals were rinsed of debris and placed in syringes with filtered seawater. Syringe volume was adjusted to the desired volume (3-7 ml), and these chambers were sealed with two-way stopcocks. Manipulations during dark conditions were done using indirect red light, after which syringes were wrapped with aluminum foil to prevent light exposure. Syringes were gently inverted and agitated prior to each end-point measurement to ensure mixing within the chambers. Oxygen concentration in each syringe was measured at the start and end of each incubation using a FireSting fiber-optic oxygen meter (Pyro Sciences) that was calibrated using air saturated seawater and zeroed using a 2% sodium sulfite solution. To control for slight variations in temperature during measurement, another unsealed syringe containing filtered seawater was used as a ‘blank’, and its value was documented simultaneously. In addition, for every three experimental syringes, a fourth chamber was filled with water but was left without an animal as a control for bacterial respiration. At the conclusion of the incubation, anemones were rinsed with distilled water, placed in pre-weighed aluminum dishes, dried at 70°C for >3 days, and weighed on a microbalance. Oxygen consumption rates were calculated based on the change in oxygen concentration and corrected for the relatively small amount of bacterial respiration (<0.5% of saturation) from the control chambers.

### Statistical analysis

For presentation (Figs 1, 2), oxygen consumption rate was normalized to dry weight. Effects of treatment groups and times on oxygen consumption rates were evaluated using analyses of covariance (ANCOVAs) with dry mass as a covariate; both the dependent variable and covariate were log-transformed. Assumptions of normality and homogeneity of variance were tested using the Shapiro–Wilk test and Levene's test, respectively. Where significant main effects were detected, Tukey's post-hoc tests were used to distinguish among groups. All statistical analyses were conducted in R (3.1.0) (http://www.r-project.org/).
